# Lysosomal storage, impaired autophagy and innate immunity in Gaucher and Parkinson's diseases: insights for drug discovery

**DOI:** 10.1098/rstb.2022.0381

**Published:** 2024-04-08

**Authors:** Alexander Hull, Magda L. Atilano, Laith Gergi, Kerri J. Kinghorn

**Affiliations:** Department of Genetics, Evolution & Environment, Institute of Healthy Ageing, Darwin Building, Gower Street, London WC1E 6BT, UK

**Keywords:** Gaucher disease, Parkinson's disease, autophagy, immunity

## Abstract

Impairment of autophagic–lysosomal pathways is increasingly being implicated in Parkinson's disease (PD). *GBA1* mutations cause the lysosomal storage disorder Gaucher disease (GD) and are the commonest known genetic risk factor for PD. *GBA1* mutations have been shown to cause autophagic–lysosomal impairment. Defective autophagic degradation of unwanted cellular constituents is associated with several pathologies, including loss of normal protein homeostasis, particularly of α-synuclein, and innate immune dysfunction. The latter is observed both peripherally and centrally in PD and GD. Here, we will discuss the mechanistic links between autophagy and immune dysregulation, and the possible role of these pathologies in communication between the gut and brain in these disorders. Recent work in a fly model of neuronopathic GD (nGD) revealed intestinal autophagic defects leading to gastrointestinal dysfunction and immune activation. Rapamycin treatment partially reversed the autophagic block and reduced immune activity, in association with increased survival and improved locomotor performance. Alterations in the gut microbiome are a critical driver of neuroinflammation, and studies have revealed that eradication of the microbiome in nGD fly and mouse models of PD ameliorate brain inflammation. Following these observations, lysosomal–autophagic pathways, innate immune signalling and microbiome dysbiosis are discussed as potential therapeutic targets in PD and GD.

This article is part of a discussion meeting issue ‘Understanding the endo-lysosomal network in neurodegeneration’.

## 1. Introduction

Autophagy is a cellular process critical to the degradation and recycling of cellular components, such as damaged organelles, misfolded proteins and other cellular waste. It involves the engulfment of unwanted cellular components into a double-membrane-bound vesicle and their breakdown following fusion with the lysosome [1]. Autophagy thus acts as a quality control system to maintain cellular homeostasis and to protect against cellular stress and damage. In addition, it also plays a pivotal role in cellular metabolism and energy production, as well as in regulation of the immune system. Defects in autophagy have been linked to numerous age-related disorders, including neurodegenerative disease, cancer and cardiovascular disease [2]. This review will focus on the role of autophagy in Parkinson's disease (PD) and neuronopathic Gaucher disease (nGD), two distinct neurodegenerative disorders sharing a genetic link through the *GBA1* gene. The association between autophagic defects and immune dysfunction that occurs in both these conditions will be explored. Immune dysregulation is increasingly being associated with pathogenic communication between the gut and brain in PD. This so-called gut–brain axis involves a bidirectional crosstalk between the gut and brain and has been implicated in both health and disease [3,4]. Although the precise relationship between these two organs is yet to be elucidated, it is likely to be complex, involving direct neuronal, endocrine, metabolic and immune system mediators [5]. Here, we will explore the evidence that autophagic defects in the gut may directly contribute to gut dysfunction and the ensuing spread of inflammation and α-synuclein (αSyn) to the brain in PD.

## 2. Autophagy

Autophagic clearance of cellular constituents can occur in both a targeted and an untargeted manner. Clearance of entire organelles or regions of the cell can be achieved via macroautophagy, which can be induced by nutrient or proteostatic stress. This involves phosphorylation of two principal initiation complexes, the ULK1 and beclin 1–VPS34, both of which are negatively regulated by mTOR and phosphorylated and activated by AMP-activated protein kinase (AMPK) [6]. Both complexes are localized to a pre-phagophore initiation site, and through a phosphorylation cascade drive formation of a double-membraned cup-like structure, the phagophore. The latter envelops cellular debris and can initiate from diverse intracellular membranes, but is principally derived from the endoplasmic reticulum (ER) or endosomal network [7]. The phagophore is released from its initiation membrane following a scission event, elongates and fully circularizes to form an autophagosome. This structure then fuses with a lysosome to form an autolysosome, either by a transient ‘kiss-and-run’ event, where lysosomal factors are transferred through a pore, or through complete fusion of the vesicles. Lysosomal hydrolases are then released, and acidification factors from the lysosomal membrane reduce intraluminal pH, allowing degradation of waste cellular components.

While macroautophagy relies on the formation of an internalizing vesicle that undergoes a series of fusion/fission events, before fusing with the lysosome, microautophagy is the product of direct internalization of the lysosome [8,9]. Sequestosome 1, p62, is a selective autophagic receptor that binds ubiquitinated substrates and delivers them to the autophagosome through interaction with LC3-II. Both p62 and LC3-II are autophagy substrates, and their turnover represents a readout of autophagic flux [10,11]. Receptors for all major forms of autophagy are upregulated alongside key lysosomal biogenesis genes as transcriptional targets of transcription factor EB (TFEB) [12].

The differential abundance, and degradation kinetics of intracellular constituents necessitate that autophagy be tailored to degradation of specific components, particularly proteins with long half-lives and amyloidogenic potential. Chaperone-mediated autophagy (CMA) accomplishes this through the use of co-chaperones such as HSC70. These recognize degradation motifs such as KFERQ on target substrates and traffic them to specific receptors tethered to the lysosomal membrane, notably LAMP2, for internalization and degradation [9].

## 3. Parkinson's disease genes map to endo-lysosomal trafficking pathways

PD is a progressive neurodegenerative disorder and the most common movement disorder, affecting approximately 1–2% of people over the age of 60 years [13]. Clinically, people with PD display both motor and non-motor symptoms. Motor problems include tremor, muscle rigidity, bradykinesia and postural instability. Non-motor features, such as gastrointestinal dysfunction, can present as many as 20 years before the onset of motor signs [14]. The neuropathological hallmark of PD is the presence of intraneuronal inclusions called Lewy bodies (LBs), composed predominantly of aggregated αSyn [15]. Most cases of PD are sporadic, being influenced by both genetic risk and environmental factors. Approximately 15% of PD cases are familial and linked to monogenic mutations in more than 20 genes [16]. Notably, most of these established disease genes map to the intracellular endosomal–lysosomal network, including autophagic and endosomal trafficking pathways [17]. For example, mutations in the *PINK1* and *PARKIN* genes cause early onset autosomal recessive PD and are associated with defects in mitophagy. The latter is the process by which defective mitochondria are cleared from the cell by macroautophagy [18,19]. Mutations in the *VPS35* (vacuolar protein sorting 35 homologue) gene have been linked to autosomal dominant PD [20]. This gene encodes a subunit of the retromer complex, a molecular sorting machine that directs specific proteins and lipids from the trans-Golgi network to endosomes for further processing, degradation or recycling [21–23]. See table 1 for a summary of known PD genes and their links to endo-lysosomal dysfunction.

**Table 1.** Parkinson's disease causal and risk genes linked to endo-lysosomal dysfunction. Autosomal dominant, AD; autosomal recessive, AR; chaperone-mediated autophagy, CMA; Lewy body, LB; early onset, EO; late onset, LO; intellectual disability, ID; autistic spectrum disorder, ASD.

gene symbol	protein name	inheritance	clinical and neuropathological features	role in endo-lysosomal network
*SNCA/PARK1*	α-synuclein (αSyn)	AD	EOPD, LB pathology	Mutant forms of αSyn inhibit CMA [24].
*PARKIN/ PARK2*	Parkin	AR	EOPD, no LB pathology	Parkin promotes mitophagy of damaged mitochondria through polyubiquitination of VDAC6 on the outer membrane [25].
*PINK1/PARK6*	serine/threonine-protein kinase	AR	EOPD, no LB pathology	PINK1 phosphorylates Parkin on the outer membrane of mitochondria to initiate mitophagy [19].
*DJ-1/PARK7*		AR	EOPD, no LB	DJ-1 regulates chaperone-mediated autophagy (CMA) [26].
*LRRK2/PARK8*	leucine-rich repeat serine/threonine-protein kinase 2	AD	LOPD, no LB pathology	LRRK2 is a kinase with a role in retromer function [27]. It is a positive regulator of autophagy through activation of AMPK [28,29].
*PLA2G6/ PARK14*	Phospholipase A_2_	AR	EO PD, dystonia parkinsonism	phospholipase A_2_/iPLA2-VIA binds the retromer subunits Vps35 and Vps26 and enhances retromer function [30].
*FBOX7/ PARK15*	F-box domain-containing protein	AR	EO PD, LB pathology	FBXO7 participates in mitochondrial maintenance through direct interaction with PINK1 and Parkin in the process of mitophagy [31].
*VPS35/ PARK17*	vacuolar protein sorting-associated protein 35	AD	LOPD, no LB pathology	Vps35 is a retromer component involved in the transport of select cargo proteins between vesicular structures such as the endosome and lysosome and the trans-Golgi network [23].
*Auxilin/ PARK19*	auxilin	AR	EOPD, LB pathology	Auxilin regulates the clathrin-mediated endocytosis pathway in neurons [32].
*DNAJC13/ PARK21*	DNAJC13	AD	LOPD, LB pathology	DNAJC13 is an endosome protein that regulates endosomal membrane trafficking. Its mutation leads to αSyn accumulation in the endosomal compartment [33].
*Rab39B*	Ras-related protein Rab-39B	X-linked	EOPD, ID, ASD, LB pathology	Rab39B is a RabGTPase localized to the Golgi; it has a proposed role in endosomal trafficking [34].
*GBA1*	glucocerebrosidase (GCase, GBA)	risk factor bi-allelic mutations cause Gaucher disease; heterozygous mutations are the most common genetic risk factor for PD [35]	LOPD, LB pathology	GBA is a lysosomal enzyme that hydrolyses glucosylceramide to ceramide and glucose. Mutations in *GBA1* cause lysosomal dysfunction [36]. Mutant GCase blocks αSyn degradation by CMA [37].
*Rab7L1/PARK16*	Rab7L	risk locus		Rab7L1 interacts with LRRK2 to modify endo-lysosomal and Golgi sorting [38].

Impairment of endo-lysosomal pathways in the cell leads to a failure to degrade misfolded and damaged proteins, particularly αSyn, which relies on degradation via CMA and macroautophagy [39]. This in turn results in the accumulation of αSyn intracellularly to form LBs [15]. The precise mechanisms by which αSyn aggregation leads to neurotoxicity are not fully understood, but include many diverse processes, such as mitochondrial dysfunction and oxidative stress, ER stress, calcium dyshomeostasis and effects on DNA and histone methylation [40]. Moreover, the accumulation of αSyn can further lead to impairment of lysosomal degradation, by disrupting the vesicular ER–Golgi trafficking of glucocerebrosidase (GCase) [35,41].

Furthermore, post-mortem evaluation of PD patient brains, with and without *GBA1* mutations, has revealed widespread GCase deficiency most pronounced in the substantia nigra (SN) [42,43]. This suggests potential therapeutic benefits from reversing defects associated with GCase deficiency in all forms of PD. Mechanistically, links between the functional loss of GCase and αSyn accumulation have been described as occurring in a bidirectional feedback loop in the synucleinopathies [36]. It was previously demonstrated in primary neuronal culture and PD mouse models that GCase deficiency potentiates αSyn pathology and that pathogenic forms of αSyn result in a reduction in GCase activity [44].

Bi-allelic mutations in the *GBA1* gene have been known for decades to cause the commonest lysosomal storage disorder (LSD) GD [45]. This is a multi-systemic metabolic disorder, characterized by the build-up of glucosylceramide (GlcCer) within macrophages, which deposit in various tissues such as the liver, spleen and bone marrow, causing tissue dysfunction and inflammation. GD is clinically subdivided according to whether there is central nervous system (CNS) involvement. Type I is the most common form and is notable for its lack of primary neurological pathology. Types II and III GD represent acute and chronic forms of nGD, respectively. Central to the pathogenesis of GD is lysosomal dysfunction. Given the role of the lysosome in several intracellular degradative processes, including the various forms of autophagy, defects in such pathways are increasingly being linked to GD [46–48].

## 4. Dysregulation of autophagic pathways in *GBA1*-associated disease

Lysosomal-autophagic dysfunction has been well described in GD and PD linked to *GBA1* mutations (GBA-PD) [46–49]. Studies in post-mortem GBA-PD brains demonstrated that mutant forms of GCase at the lysosomal surface impair CMA degradation of αSyn [37]. Using dopaminergic-like cell lines expressing two different *GBA1* mutant constructs (N370S and L444P) on a background of *GBA1* knockout, it was shown that the earlier steps of autophagosomal formation are upregulated, with elevated levels of phosphorylated ULK1, an upstream initiating factor for autophagic activation. Despite this, PI3K complex components such as beclin, regulating phagophore formation, were not increased. These findings likely represent an early compensatory response to the partial loss of normal autophagic–lysosomal degradation [50].

Further upstream, mTOR phosphorylates the CLEAR (Coordinated Lysosomal Expression and Regulation) network activator TFEB to prevent its nuclear export, thus repressing transcription of autophagic–lysosomal genes. Accordingly, in a fly model of nGD, mTOR activity was dysregulated and *MITF*/*TFEB* was upregulated, in response to a block in lysosomal–autophagic function [48,49]. GD induced pluripotent stem cell (iPSC)-derived neuronal progenitors and differentiated neurons also displayed evidence of mTORC1 hyperactivation and increased TFEB phosphorylation. Moreover, pharmacological inhibition of glucosylceramide synthase reversed mTORC1 hyperactivation, implying that the abnormal mTORC1 activity is mediated by the build-up of glycosphingolipids in GD cells [51].

In keeping with the autophagy impairments in GD, increased LC3-II/LC3-I ratios and p62 accumulation, both consistent with a defect in autophagosome–lysosome fusion, have been observed in mouse [52], patient iPSC-derived neuronal models [50] and fly models of nGD [48,49]. Moreover, studies in iPSC-derived midbrain dopaminergic neurons from patients with *GBA1* mutations demonstrated an increase in both LC3 and LAMP1, with a low co-localization index, suggesting a block specifically at the fusion stage of autophagy [53].

Additionally, LAMP2 is upregulated in a neuronopathic-like GD *saposin C*/*Gba1 V394L* double mutant mouse model, reflecting either increased chaperone-mediated autophagy (CMA) or simply upregulation of CLEAR network genes [52]. Further probing of these mice demonstrated both a significant reduction in LAMP2 specifically in lysosomal membrane fractions, and a decrease in CMA-mediated proteolysis. CMA was also impaired in human *GBA1* mutant neuronal cell lines in association with intralysosomal accumulation of sphingolipids [50]. These findings suggest that CMA is not inhibited through direct lysosomal dysfunction, but rather via destabilization of membrane microdomains as a result of the lipid dyshomeostasis that occurs in GCase deficiency. It is also possible that this mechanism could additionally disrupt autophagosome–lysosome fusion.

*GBA1* mutations have also been linked to impairment of mitophagy. Hippocampal neurons from knock-in mice harbouring heterozygous L444P *GBA1* mutations display impairments in autophagy and mitochondrial priming, both necessary for mitophagy [54]. These defects were shown to be dependent on gain-of-function mechanisms, as expression of L444P mutant GBA in a neuronal cell line resulted in similar defects in mitophagy despite intact GCase activity. Consistent with this, examination of post-mortem brain tissue from individuals with GBA-PD revealed mitochondrial abnormalities and impaired autophagy. Moreover, complete genetic depletion of GCase activity led to a block in the lysosomal clearance of autophagic cargo.

In addition to impairment in macroautophagy and CMA, autophagic lysosomal reformation (ALR) has also been shown to be compromised in mutant *GBA1* primary mouse neurons and patient-derived fibroblasts [55]. ALR is regulated by mTOR and involves the regeneration of functional lysosomes from autolysosomes established during autophagy [56]. Defects in this process lead to a failure to maintain a pool of mature and functional lysosomes crucial for the degradation of misfolded proteins such as αSyn. Moreover, it is possible that this process may reflect a common pathology in PD, especially given the fact that GCase deficiency and lysosomal dysfunction occur both with ageing and in sporadic PD [43,50,57–59].

Thus the exact mechanisms underlying the autophagic defects that occur in the context of *GBA1* mutations remain elusive, and several differing mechanisms may be involved. It is possible that endosomal and autophagic defects due to *GBA1* loss-of-function may affect multiple points of the endo-lysosomal network, simultaneously or in a stepwise manner, with initial lysosomal dysfunction culminating in a block in autophagosome–lysosomal fusion and impairment of multi-vesicular body (MVB)/autophagosome maturation [53]. Moreover, downstream of lysosomal defects, localized macromolecular deficiencies due to inhibition of the lysosomal ceramide salvage pathway may impinge on normal vesicular functions. Furthermore, as macrophages transition to Gaucher cells, their capacity to remove toxins from the circulation may be diminished. Accordingly, circulating glucosylsphingosine (GlcSph) is elevated in GD and PD patients [60]. A recent study showed GlcSph levels are raised in multiple brain regions in both idiopathic and *GBA1*-linked PD patients, demonstrating a positive correlation with αSyn pathology [61]. GlcSph is formed by deacylation of GlcCer by the lysosomal enzyme acid ceramidase and high levels of this sphingolipid have been identified in nGD brains. Elevated levels of GlcSph activate mammalian mTORC1, interfering with lysosomal biogenesis and autophagy in nGD patient iPSC-derived neurons [62].

## 5. Autophagic dysregulation in Parkinson's disease

*α*Syn is a CMA-cargo bound by the cytosolic chaperone HSC70 and internalized by LAMP2A. The pro-aggregatory A30P and A53T SNCA (αSyn) mutations that increase PD risk reduce CMA degradation, and allow these mutant proteins to accumulate and form more toxic conformers [24]. LRRK2, mutations in which cause autosomal dominant PD, is a CMA substrate. The most common PD-linked variant, G2019S, not only abrogates CMA-mediated uptake of LRRK2, but can dominantly disrupt the LAMP2A complex and interfere with CMA globally [63]. Changes in secondary structure of autophagy substrates are not the only CMA defect evidenced in PD; LAMP2A and HSC70 are both decreased in the brains of people with PD [64]. Furthermore, boosting CMA through lentiviral LAMP2A administration has been shown to reduce αSyn neurotoxicity in neuronal cell culture and in a rat synucleinopathy model [65].

While monomeric αSyn is degraded through CMA, higher-order aggregates are substrates for macroautophagy. Insoluble aggregates persist in autophagosomes following the degradation of soluble αSyn and lead to a build-up of early-stage autophagosomes upstream of the fusion step with the lysosome [66]. Overexpression of wild-type αSyn results in an indirect block to macroautophagy, contingent on inhibiting Rab1a-mediated trafficking of Atg9, a pre-autophagosomal regulator [67]. In *Drosophila* models overexpressing human αSyn, autophagosome–lysosome fusion is blocked through aberrant actin stabilization, resulting from an interaction between αSyn and spectrin [68]. Thus, although autophagic defects can drive αSyn accumulation, so too can the reverse occur, with the abnormal build-up of αSyn causing autophagic defects. Irrespective of the precise temporal relationship between αSyn aggregation and autophagy dysfunction, stimulating autophagy to increase the degradation of misfolded αSyn offers a potential therapeutic avenue in PD.

In addition to the degradation of misfolded proteins, autophagy is also responsible for the removal of defective organelles within the cell, including abnormally depolarized mitochondria. Elegant work in *Drosophila* unravelled the role of two key players in mitophagy, a mitochondria-specific form of autophagy. Subsequent studies have further refined the role of these two PD-associated proteins, PINK1 and Parkin, as functioning in the ubiquitinating pathway to target damaged mitochondria for degradation. Indeed, defects in mitophagy are a hallmark of PD, wherein accumulation of damaged mitochondria and subsequent oxidative stress are present in multiple PD models [25,69,70].

## 6. Autophagy dysfunction and its link to innate immune dysregulation in Parkinson's disease and Gaucher disease

Defects in autophagy can lead to dysregulation of both innate and adaptive immunity [71,72]. Thus, the autophagic impairment observed in both PD and GD may contribute to immune activation and neuroinflammation. In PD, there is evidence that microglia, innate immune cells of the brain, are activated through pattern recognition receptors, such as Toll-like receptors (TLRs), which recognize specific molecular damage- and pathogen-associated motifs [73]. Indeed, toxic oligomeric forms of αSyn released by neurons interact with TLR-2 on the surface of microglia, leading to their activation [74]. Accordingly, TLR-2 levels are increased in post-mortem PD brains, correlating with the αSyn pathology burden [75]. TLR-2 inhibits neuronal autophagy through regulation of the AKT–mTOR pathway, resulting in the accumulation of αSyn aggregates [76].

Autophagy also plays a role in innate immune responses in GD. A link between lysosomal function and inflammasome activation has been demonstrated in GD macrophages [46]. Impaired autophagy in these cells leads to an increase in the autophagy adaptor protein p62, resulting in activation of p65–NF-κB in the nucleus and secretion of pro-inflammatory cytokines, such as interleukin (IL)-β and IL-6. Work in a fly model of *GBA1/Gba1* knockout has shown autophagic–lysosomal defects in the gut wall, in association with gut dysfunction and intestinal microbiome dysbiosis [49]. Moreover, stimulation of autophagy, or eradication of the intestinal microbiome, by raising flies under germ-free conditions, leads to partial rescue of both lifespan and locomotor defects. These effects are yet to be confirmed in patients with *GBA1*-linked disease, but allude to the fact that gut dysfunction, particularly intestinal autophagy defects, may contribute to the development of PD in *GBA1* mutation carriers and individuals with GD. A study using a murine model revealed that impaired microglial autophagy increases neuroinflammation in an NLRP3 inflammasome-dependent manner, leading to motor and cognitive impairments [77]. Previous studies in *Drosophila* have shown how a block in autophagy can lead to unregulated immune activation. It was demonstrated that the NF-κB immunodeficiency (IMD) innate immune pathway, analogous to mammalian TIR-domain-containing adapter-inducing interferon-β (TRIF) signalling, is regulated by selective autophagy. Baseline degradation of IMD pathway signalling complexes regulates this immune response and prevents deleterious constitutive IMD activation [78]. It was also found that selective autophagic degradation of the IKK complex in *Drosophila* is mediated by the interaction of Kenny with Atg8a, inhibiting IMD pathway activation by commensal bacteria [79].

## 7. Innate immune activation and inflammation in Parkinson's disease and Gaucher disease

Genetic, clinical and pre-clinical studies in animal models highlight involvement of both the innate and adaptive immune system in PD and GD, both centrally in the brain and peripherally [80–84]. Recent genome-wide association studies have identified more than 90 genetic risk loci for PD, accounting for 16–36% of its heritability [85,86]. Pathway-based analyses of genetic risk loci have shown gene enrichment for biological pathways related to immune function [87]. Several immune-related genetic variants have been linked to PD risk, including at the human leucocyte antigen (HLA) [88], tumour necrosis factor (TNF)-α [89] and TLR-9 [90] loci. From a clinical perspective, elevated levels of proinflammatory cytokines have been observed in the serum, brain and cerebrospinal fluid (CSF) of PD patients [91–96]. For example, increased levels of IL-2, IL-6 and TNF-α, as well as the chemoattractant protein (MCP-1), were increased in the CSF of PD patients [92,93,96].

There is mounting evidence to support a link between αSyn and immune activation in PD. Nigrostriatal pathway injection of lipopolysaccharide (LPS) into transgenic mouse models with normal or increased levels of αSyn resulted in degeneration of dopaminergic neurons in the SN [97,98]. Several studies have suggested that αSyn is a damage-associated molecular pattern (DAMP) that can induce production of inflammatory cytokines in microglia [99–101]. Microglial activation and recruitment of glia to areas predisposed to dopaminergic neuronal loss are observed in post-mortem PD brains [102]. Consistent with this, positron emission tomography (PET) imaging studies have shown early microglial activation in PD brains in both cortical and subcortical areas [103]. Similar studies have also revealed microgliosis in the brains of *GBA1* mutation carriers in the absence of PD [104]. Moreover, work in primary rat and mouse midbrain cultures revealed that aggregated oligomeric αSyn is capable of activating microglia [105]. Elevated levels of TLR2 and TLR4 expression are observed in brain regions pathologically affected by PD (i.e. SN pars compacta and putamen) and in myeloid cells of the post-mortem brains of PD patients [75,106]. Kim *et al*. [74] revealed that medium containing extracellular oligomeric αSyn activates microglia and stimulates proinflammatory cytokine/chemokine production in a TLR2-dependent manner [74]. Moreover, inhibition of TLR2 in murine microglia, either through the depletion of the *Tlr2* gene or through TLR2-blocking antibodies, resulted in the elimination of proinflammatory cytokine upregulation. In a follow-up study, Kim and colleagues [107] exposed rat primary neurons in culture to murine microglia and αSyn [107]. *Tlr2* gene depletion in the microglia completely abrogated microglial neurotoxicity, typically triggered by the neuronally released oligomeric αSyn, whereas the ability of LPS to trigger microglial neurotoxicity was unaltered. These results suggest that TLR2 activation of microglia by aggregated forms of αSyn drives dopaminergic degeneration, possibly through the production of toxic agents, such as proinflammatory cytokines. In addition, TLR4 has been shown to mediate αSyn-induced murine microglial activation, phagocytosis, proinflammatory cytokine production and reactive oxygen species (ROS) production [108].

Alterations in adaptive immunity are also implicated in PD and are reviewed in detail elsewhere (see [83,109]). For example, T-cells are activated by αSyn and can infiltrate the CNS to stimulate neuroinflammation. Indeed, it was shown that overexpression of αSyn in the midbrain of mice leads to upregulation of the major histocompatibility complex (MHC) II on CNS myeloid cells, as well as infiltration of interferon (IFN)-γ secreting CD4 and CD8 T-cells into the brain [110]. Moreover, genetic deletion of CD4 resulted in dampening down of this neuroinflammatory response.

Immune system abnormalities also occur in GD, with an increased susceptibility to infection and certain malignancies [111,112]. Gene expression analysis of cultured fibroblasts from GD patients has shown gene enrichment for immune response pathways [113]. Accumulation of GlcCer in GD leads to macrophage activation, and increased expression and secretion of serum cytokines, including TNF-α, IL-1β, IL-6, IL-10 and others [80,81]. Multiple studies in mouse models of nGD show microglial and astrocytic activation, as well as infiltration of immune cells into the brain [114,115]. These pathologies are associated with widespread upregulation of pro-inflammatory cytokines, including IL-1β and TNF-α, and loss of blood–brain barrier integrity [116]. Moreover, targeted rescue of *Gba1* in the microglia and neurons of *Gba1*-deficient nGD mice leads to the reversal of GlcCer and GlcSph accumulation, reduced neuroinflammation and improved survival [115]. Furthermore, a chronic reduction of GCase in mice using an irreversible inhibitor, conduritol-β-epoxide, was associated with widespread neuroinflammation and complement C1q activation, in addition to αSyn pathology and neuronal loss [117,118]. Similar neuropathology was also reported in a zebrafish *GBA1* knockout model, which lacks endogenous αSyn. Microglial reaction in the brain was accompanied by a significant loss of dopaminergic neurons and age-dependent locomotor decline [119].

## 8. Gut-specific autophagy defects in Parkinson's disease and their effects on gut–brain axis communication

Gastrointestinal dysfunction is increasingly being implicated in PD and is consistent with the histopathological abnormalities observed in the intestinal tissue of PD patients. Braak and colleagues demonstrated Lewy body pathology in the myenteric nervous system prior to that seen in the SN [120]. It has thus been hypothesized that the gut may act as the initiating site of PD pathology. Subsequent histopathological studies on gastrointestinal tissues have demonstrated significantly greater αSyn deposition in PD patients compared with healthy controls [121,122]. Moreover, *in vivo* studies in mammalian models have shown that αSyn injected into the intestinal wall can be retrogradely transported from the myenteric plexus in a ‘prion-like’ manner via the vagus nerve to the brainstem [120,123–125], confirming a direct neuronal link between the gut and brain. Consistent with this, truncal vagotomy reduces the risk of developing PD [126].

In keeping with αSyn deposition in the gut, intestinal autophagy defects are increasingly being linked to PD. The observation of αSyn in the small intestine of healthy individuals, albeit to a lesser extent than in those with PD, suggests that the presence of low levels of aggregatory protein in the gut are insufficient to cause PD. It is thus possible that defects in the degradative pathways of the gut cells, which target the clearance of αSyn, may contribute to its abnormal accumulation within the intestinal tissue. Indeed, epigenetic inactivation of the autophagic–lysosomal pathway in the appendix tissue of individuals with PD has been observed, as evidenced by aberrant and widespread hypermethylation of autophagic–lysosomal pathway genes, similar to that seen in the brains of individuals with PD [127]. Autophagic impairment in the gut has been shown in *Gba1* knockout flies in association with increased intestinal transit time, increased intestinal wall leakiness and elevated NF-κB signalling in gut cells, as well as increased intestinal microbiome load and dysbiosis [49].

There is growing evidence to suggest that intestinal dysfunction can influence the brain via gut–brain axis communication. In accordance with gastrointestinal symptoms being an almost universal feature in PD, intestinal inflammation and an altered composition of the intestinal flora, known as the gut microbiome, have been described in PD patients [128,129]. Changes in relative bacterial abundance were observed at the phylum level between PD patients and healthy controls [130,131]. Overall, the microbiota of healthy patients was associated with low levels of LPS and flagellin [132], while the microbiome of PD patients appeared more similar to that observed in patients with inflammatory bowel disease. A study in a Taiwanese cohort revealed that alterations in the microbiota of individuals with PD are correlated with the severity of motor dysfunction, as well as increased levels of the proinflammatory cytokines IFN-γ and TNF-α in the plasma. mRNA expression levels of proinflammatory cytokines, such as TNF-α, IL-6, IL-1β and IFN-γ are significantly elevated in the ascending colon of PD patients versus controls, but decline over the course of the disease progression [128]. A meta-analysis examining the association of *Helicobacter pylori* gut infections with PD found that the presence of this bacterium is more frequent in PD patients compared with controls, and that these infections correlate with more severe motor defects [133]. Studies of two different cohorts—one in Taiwan and one in the USA—found an association between the diagnosis of inflammatory bowel disease and the risk of developing PD [134,135]. These findings indicate that intestinal inflammation may occur early in PD and may be linked to gut dysbiosis.

Sampson *et al*. [136] demonstrated in αSyn-overexpressing (ASO) mice that eradication of the intestinal microbiome resulted in the amelioration of neuropathology and locomotor dysfunction [136]. Similar results were seen in the ASO mice raised under germ-free conditions or treated with a cocktail of antibiotics postnatally. Similarly, Atilano *et al*. [49] showed that elimination of the intestinal microbiome, by raising *Gba1* knockout flies under germ-free conditions, reduces brain glial activation and decreases gut and systemic NF-κB signalling, in addition to promoting survival and locomotor performance [49]. Together these findings support a communication between the gut and brain and suggest that modulating the gut microbiome may represent a possible therapeutic avenue in PD.

## 9. Targeting lysosomal–autophagic defects and downstream pathologies to treat Parkinson's disease and Gaucher disease

### (a) Targeting autophagy

Growing evidence suggests that lysosomal–autophagic dysfunction plays a central role in PD and GD. Thus, the autophagy pathway and its regulator, the mTOR complex, represent potential therapeutic targets for the treatment of PD and *GBA1*-associated neurodegeneration. Indeed, treatment with the mTOR inhibitor Torin1 was found to enhance lysosomal biogenesis and improve autophagic clearance in GD neurons [51]. Additionally, the mTOR inhibitor rapamycin alleviated cell death in both mice and neuronal cell cultures treated with the dopamine agonist MPTP [137]. Overexpression of 4E-BP, which is negatively regulated by mTOR, or treatment with rapamycin, suppressed dopaminergic cell death in *PINK1*/*Parkin* mutant fly models [138]. Rapamycin has been shown to increase autophagy and reduce neuronal loss in a mouse model of Alzheimer's disease [139], as well as alleviating motor function in rodent PD models [140]. In a fly model of nGD, rapamycin reduced innate immune signalling in the gut and other tissues via direct stimulation of autophagy [49]. There were no significant effects on innate immunity in control flies.

As rapamycin functions upstream of autophagic initiation, it is unknown how increased autophagosomal biogenesis might rescue defects in autophagosomal–lysosomal fusion, which are characteristic of *GBA1*-depleted cells [53]. The actions of rapamycin would be predicted to increase immature autophagosomes and might therefore be expected to further stress remaining functional lysosomes. It is possible that rapamycin functions as a dual stimulator of autophagosome and lysosomal biogenesis and bypasses intermittent fusion defects as far as cellular energetics will allow. Macroautophagic induction through beclin administration, a manipulation that increases early phagosome maturation, ameliorated PD pathology in a transgenic mouse model [141]. These benefits are also interesting given the fact that beclin upregulation would not be expected to improve defects in autophagosome–lysosome fusion.

In humans, the principal therapeutic purpose of rapamycin (also known as sirolimus) is as an immunosuppressant. It suppresses the adaptive immune system and limits the proliferation of T-cells, through inhibition of S6K phosphorylation and the cdk2–cyclin complex, both of which are required for G1/S phase transition and subsequent T-cell division [142]. Inflammation and immune dysfunction are increasingly being linked to PD and GD. Therefore, rapamycin may offer dual therapeutic benefits in these disorders by virtue of its ability to directly stimulate autophagy and suppress immune activation. Another candidate therapeutic that targets mTOR is RTB101, which is currently in a Phase Ib/IIa clinical trial in combination with sirolimus in PD patients [143].

Pioglitazone, a thiazolidinedione (TZD) that is used in the treatment of type 2 diabetes, has also shown promise for the treatment of PD. Its use is associated with a lower risk of developing PD in diabetic populations [144,145]. Pioglitazone improved lysosomal–autophagic dysfunction in a fly model of *Gba1* deficiency, with return of the levels of the autophagic marker Ref(2)P/p62 towards normal [146].

### (b) Targeting GCase

Decreased levels of GCase have been demonstrated in PD and ageing human brains [59,147]. Systemic reduction of GCase activity in mice, using an irreversible inhibitor, is associated with increased inflammation, complement activation and accumulation of αSyn [117,118], thus supporting a role of glycolipid dysregulation in inflammation. Appropriately, enzyme-replacement therapy (ERT) to deliver GCase protein, and substrate-reduction therapy (SRT) to reduce the production of GlcCer and GlcSph, are already established in the treatment of GD [148]. Although these treatments are effective in protecting against non-neuropathic symptoms, most of them do not cross the blood–brain barrier (BBB). Those that do permeate the BBB have reduced efficacy and are thus not suitable to adequately treat the neuropathology associated with GD or GBA-PD [149–151]. Recently, gene therapy has been used to directly deliver a functional *GBA1* gene into the nervous system of various genetic mouse models of GD and GBA-PD [152,153]. Intracerebral adenovirus (AAV)–*GBA1* injections in αSyn-expressing (ASO) mice increase GCase protein levels and activity in various brain regions and alleviate neuroinflammation. These improvements are also accompanied by a reduction in αSyn pathology [154]. Preclinical studies in mouse models with PR001, an AAV9 vector-based gene therapy designed to deliver the *GBA1* gene directly to the CNS, revealed broad vector distribution with significant elevation of GCase levels and no adverse findings or evidence of toxicity. PR001 is currently in Phase I/II clinical trials in GBA-PD patients [155].

Alternative therapeutic strategies for GD and GBA-PD include the use of small molecules that can pass the BBB to help traffic misfolded GCase to the lysosome, increasing lysosomal GCase activity levels. One such candidate is ambroxol, a small molecule chaperone that binds mutant misfolded GCase protein in the ER to increase its trafficking to the lysosome. Ambroxol has shown promising results in GBA-PD cellular, fly and mouse models [156–160] and is now in phase II clinical trial in GBA-PD (AiM-PD study). Another approach, using molecular chaperones, is that promoting the degradation of GCase. Heat shock protein 90 (Hsp90), together with heat shock protein Hsp27, is responsible for the degradation of misfolded GCase protein. Limiting the proteasomal degradation of GCase, using specific HSP and histone deacetylase inhibitors, has shown therapeutic potential in pre-clinical studies [161,162].

### (c) Targeting the innate immune system and neuroinflammation

Given the overwhelming evidence for immune system activation in PD and GD, researchers have begun probing the therapeutic potential of anti-inflammatory and immunosuppressant drugs in PD, which modulate the immune system and inflammatory processes [109]. A number of epidemiological studies analysed in a meta-analysis have shown lowered risk of PD associated with non-steroidal anti-inflammatory drug use [163]. However, this association was not supported in all analyses [164,165]. Several studies have also examined the effect of immunomodulatory drugs on PD risk. One population-based case control study demonstrated an association between corticosteroid use and a lower risk of PD [166]. To date, despite many clinical trials involving anti-inflammatory and immunomodulatory drugs, the outcome of such studies has been disappointing [109]. Larger well-designed clinical studies are now required to fully explore the potential of modulating the immune system and inflammation in PD. Azathioprine, a licensed immunosuppressant medication, is currently being tested in a proof-of-concept randomized double-blind placebo-controlled phase II clinical trial (AZA-PD) [167].

Novel therapeutic avenues are now required, focusing on the modulation of the most harmful components of the immune system in PD. As discussed above, data from PET studies and experimental findings from animal models demonstrate that the microglial response in PD occurs early, often preceding neuronal loss [104,168,169]. Specific aggregated conformations of αSyn accumulate with disease progression, and impair microglial phagocytosis [170]. Indeed, it has been postulated that as PD progresses, microglial activity may shift away from a neuroprotective role toward a more neurotoxic one [168,169]. This seemingly dual neuroprotective and neurodegenerative capacity of microglia in the PD brain suggests that novel immunomodulatory therapies should be targeted to the optimization of the neuroprotective and the downregulation of neurotoxic activities, as opposed to generic suppression of microglial function. For example, in a mouse model of multiple system atrophy, a synucleinopathy, a selective TLR4 agonist promoted microglial clearance of αSyn, while preventing αSyn-mediated TLR4 upregulation of proinflammatory cytokine secretion, resulting in neuroprotection and improved disease outcomes [171]. Therefore, proteins such as TLR4 and TLR2, which have been identified as contributors to neuroprotective and neurodegenerative activities, may represent potential therapeutic targets in PD and GD.

Moreover, useful therapeutic insights may be gained by further studying the immunomodulatory effects of compounds that have shown benefit in animal models of PD. Interestingly, resveratrol, a naturally occurring phenol present in the skin of grapes and other foods, ameliorated dopaminergic neuronal degeneration and pro-inflammatory cytokine production in a 6-hydroxydopamine (6-OHDA)-provoked rodent model [172]. In another study, resveratrol enhanced the degradation of αSyn in a PC12 cell line via the induction of autophagy [173].

### (d) Targeting the intestinal microbiome

Microbiome dysbiosis is likely a key driver of neuroinflammation in GD and PD. Elimination of gut microbiota with antibiotics reduced neuroinflammation in a fly model of nGD [49] and a mouse model of PD [136]. Thus, novel antibiotic regimes targeting the gut microbiome may represent potential treatments for PD. Indeed, tetracycline and its derivatives have been associated with both anti-inflammatory and anti-apoptotic activities in *in vitro* and *in vivo* models of PD [174]. In a cell culture study, minocycline reduced neuronal cell death by inhibiting the activation and proliferation of microglial cells via inhibition of NMDA-induced activation of p38 MAPK [175]. In addition, minocycline supressed microglial activation and expression of proinflammatory factors in a 1-methyl-4-phenyl-1,2,3,6-tetrahydropyridine (MPTP)-mouse model of nigrostriatal dopaminergic neuronal degeneration [176]. Doxycycline is currently one of the most widely used antibiotics in the world, is highly effective and is inexpensive with minimal clinical side effects. Several studies have demonstrated the neuroprotective effects of this antibiotic on dopaminergic neurons. Systemic doxycycline treatment in a 6-OHDA mouse model of PD resulted in decreased microglial activation [177]. In primary microglia incubated with LPS, the use of this antibiotic led to decreased microglial activation and reduced expression of inflammatory mediators such as TNF-α and ROS. These effects were facilitated through the inhibition of the p38 MAPK and NF-κB signalling pathway. Doxycycline treatment also inhibited degeneration of LPS-induced dopaminergic neurons through downregulation of the MHC-II [178].

Despite these studies, the neuroprotective effect of minocycline and doxycycline in neurodegenerative models has proven to be variable and sometimes contradictory. The results obtained from clinical trials are inconclusive and do not support their current use in PD. Short-, medium- and long-term antibiotic use is linked to a plethora of effects on gut microbiota, including reduced species diversity, altered metabolic activity, the emergence of antibiotic-resistant organisms and recurrent intestinal infections. Moreover, adverse harmful effects may occur in the context of specific diseases [179]. For example, harmful neurological clinical outcomes were observed in a clinical phase III trial investigating the effect of minocycline in amyotrophic lateral sclerosis (ALS) patients [180]. Interestingly, chemically modified tetracycline, lacking antibacterial activity, has been shown to reduce microglial activation and secretion of pro-inflammatory cytokines in cell culture on exposure to neurotoxic αSyn aggregates [181], highlighting the potential therapeutic use of modified antibiotic agents in neurodegeneration.

The previously mentioned study by Sampson and colleagues observed that colonization of germ-free ASO mice with microbiota from PD patients resulted in enhancement of locomotor defects compared with microbiota transplants from healthy human donors and was associated with a reduction in Lachnospiraceae and Ruminococcaceae bacterial genera [136]. Following on from this, a preliminary study on a small group of people with PD revealed that faecal microbiota transplant (FMT) resulted in the improvement of both motor and non-motor symptoms at six months [182]. Further, larger studies are now required to examine the potential therapeutic benefits of FMT in PD in the longer term.

### 10. Concluding remarks and future directions

PD is often considered an αSyn-centric disease, with LB-containing aggregated forms of this protein representing the neuropathological hallmark [15]. However, diverse *Drosophila* models of PD, including those modelling *PINK1*, *PARKIN*, *PLA2G6*, *LRRK2* and *GBA1* mutations, display PD-like neurodegenerative phenotypes, despite the absence of endogenous αSyn [19,48,183–185]. Therefore, perhaps it is useful in terms of our understanding and development of therapeutic strategies, that PD is considered primarily a disorder of proteostasis, with underlying impairments in endo-lysosomal autophagic pathways.

There is a wealth of evidence to support a therapeutic role of targeting autophagy in all forms of PD. More generally, loss of protein homeostasis is a key hallmark of ageing [186], and therapies targeting the autophagic pathway, notably rapamycin, are known to extend lifespan across multiple organisms [187–189]. As neurodegeneration is likely mechanistically intertwined with the ageing process, it is probable that there will be some intersection between treating ageing and PD.

As we have discussed, defects in autophagy, which are common to both PD and GD, can trigger innate immune responses [190]. However, a precise understanding of the nature of immune activation, involving both innate and adaptive branches, and their relationship to autophagic–lysosomal defects across disease progression in PD and GD is lacking. Data from a fly model of nGD suggest that immune activity is driven by autophagic defects in various tissues, including the gut, and that reversing autophagic impairment with the mTOR inhibitor rapamycin reverses the innate immune signalling in association with increased lifespan and other health benefits [49]. Thus, rapamycin may represent a potential therapy for the treatment of *GBA1*-related neurodegeneration and other forms of PD characterized by lysosomal–autophagic impairment. More broadly, unravelling the temporal relationship between autophagic–lysosomal dysfunction, peripheral and central immune activation, gut dysfunction, and microbiome alterations, as well as defined neuropathologies, will be crucial to identifying new therapeutic avenues for the treatment of these diseases. The questions of how, precisely, the immune system and inflammation contribute to neurodegeneration, and whether different stages of PD progression are characterized by differing immune responses, both neuroprotective and neurotoxic, are yet to be answered.

Accordingly, focused studies on immune changes and autophagic–lysosomal integrity across different tissues over the course of the disease are required. Such studies will benefit from appropriate animal models and access to human subjects and tissues throughout the natural history of the disease, including premotor stages. These approaches will profit from non-invasive techniques, such as PET imaging modalities [103,191,192] to study neuroinflammation, in combination with sequential analysis of readily available patient samples, including peripheral blood and CSF. Together with transgenic animals modelling αSyn neurotoxicity and familial PD linked to mutations in genes functioning in endo-lysosomal–autophagic processes, a detailed temporal exploration of the immune landscape in PD will be possible. Moreover, advances in single cell 'omics, including transcriptomic, proteomic and lipidomic analyses, will enable deep profiling of changes at the cellular and molecular levels in immune signalling and autophagic–lysosomal machinery.

Such knowledge will enable the identification of new therapeutic targets and strategies aimed at diverse pathologies, as well as elucidation of the optimal therapeutic windows for such interventions. The latter is an important consideration in the PD research field, as it is likely the reason why most clinical trials of disease-modifying PD therapies have failed. The prodromal or premotor stage of PD occurs between 5 and 20 years before the onset of motor symptoms, when 50–60% of dopaminergic neurons are already lost within the SN [193]. Thus, the potential of therapeutic drugs to prevent neuroinflammatory and neurodegenerative processes is significantly limited in diagnosed PD patients who are already manifesting motor symptoms. Early recognition of premotor PD will be critical to initiating possible neuroprotective therapies at a stage when such interventions are likely to be most effective. An increasing number of markers with sufficient evidence to warrant their inclusion in prodromal PD research criteria have arisen in recent years, and include premotor clinical, tissue, fluid and neuroimaging biomarkers [193].

**Figure 1.**
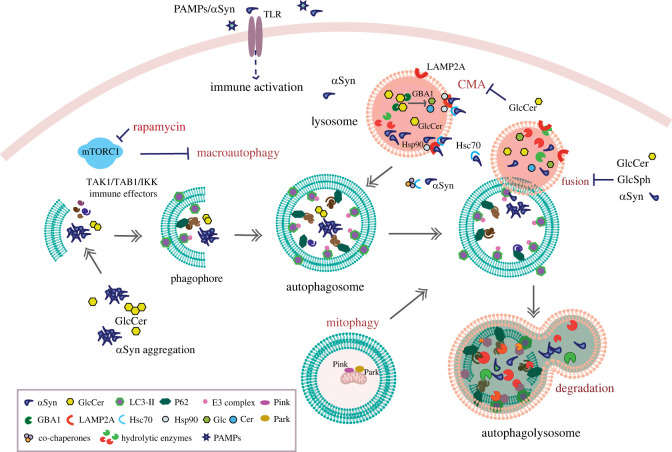
Schematic summarizing the key interactions between molecular hallmarks of *GBA1*-associated disease, the autophagic–lysosomal system and innate immune pathways.

Finally, studies will need to take into consideration the substantial evidence that PD pathogenesis may begin in peripheral tissues such as the gut. This highlights the potential benefits of developing therapies targeted at autophagic–lysosomal, immune and related pathologies in these tissues. Future therapeutic strategies, as well as perhaps being targeted at non-neuronal tissues, may also involve combinational therapies, targeting separate pathologies simultaneously or at various stages of disease progression, to reverse and prevent the development of PD and GD (figure 1).

## Data Availability

This article has no additional data.
